# Non-Emergency Medical Transportation Needs of Middle-Aged and Older Adults: A Rural-Urban Comparison in Delaware, USA

**DOI:** 10.3390/ijerph14020174

**Published:** 2017-02-10

**Authors:** Matthew Lee Smith, Thomas R. Prohaska, Kara E. MacLeod, Marcia G. Ory, Amy R. Eisenstein, David R. Ragland, Cheryl Irmiter, Samuel D. Towne, William A. Satariano

**Affiliations:** 1 College of Public Health, The University of Georgia, Athens, GA 30602, USA; 2 Texas A&M School of Public Health, Texas A&M University, College Station, TX 77844, USA; mory@sph.tamhsc.edu (M.G.O.); towne@sph.tamhsc.edu (S.D.T.Jr.); 3 College of Health and Human Services, George Mason University, Fairfax, VA 22030, USA; tprohask@gmu.edu; 4 Fielding School of Public Health, University of California, Los Angeles, CA 90095, USA; kara.e.m@gmail.com; 5 Feinberg School of Medicine, Northwestern University, Chicago, IL 60209, USA; amy.eisenstein@northwestern.edu; 6 School of Public Health, University of California, Berkeley, CA 92521, USA; davidr@berkeley.edu (D.R.R.); bills@berkeley.edu (W.A.S.); 7 SafeTREC, University of California, Berkeley, CA 92521, USA; 8 Easter Seals, Chicago, IL 91106, USA; cirmiter@sbcglobal.net

**Keywords:** rural, non-emergency medical transportation, transportation, travel distance, healthcare access, United States of America, aging

## Abstract

*Background*: Older adults in rural areas have unique transportation barriers to accessing medical care, which include a lack of mass transit options and considerable distances to health-related services. This study contrasts non-emergency medical transportation (NEMT) service utilization patterns and associated costs for Medicaid middle-aged and older adults in rural versus urban areas. *Methods*: Data were analyzed from 39,194 NEMT users of LogistiCare-brokered services in Delaware residing in rural (68.3%) and urban (30.9%) areas. Multivariable logistic analyses compared trip characteristics by rurality designation. *Results*: Rural (37.2%) and urban (41.2%) participants used services more frequently for dialysis than for any other medical concern. Older age and personal accompaniment were more common and wheel chair use was less common for rural trips. The mean cost per trip was greater for rural users (difference of $2910 per trip), which was attributed to the greater distance per trip in rural areas. *Conclusions*: Among a sample who were eligible for subsidized NEMT and who utilized this service, rural trips tended to be longer and, therefore, higher in cost. Over 50% of trips were made for dialysis highlighting the need to address prevention and, potentially, health service improvements for rural dialysis patients.

## 1. Introduction

### 1.1. Rural Health

It is estimated that over 20% of the American population resides in rural areas and this population is distributed over 80% of the U.S. land area [1]. The proportion of the U.S. population that is rural anticipated to decrease, however, projections indicate growth in some rural areas with an increase in racial and ethnic diversity in forthcoming years [2]. Further, rural areas contain larger proportions of older adult inhabitants (i.e., individuals aged 65 years and older) compared to urban areas [3,4]. Rural-based disparities have been widely recognized in recent decades, with these populations carrying disproportionate burdens in terms of chronic conditions, poverty, poor health outcomes, occupational hazards, and geographic isolation [5,6,7,8].

While rural populations often encounter similar health-related challenges as those living in urban or suburban areas, these problems are often intensified and may be attributed to limited access to healthcare and community resources [2,5,9,10]. Health-related resources are more difficult to obtain in rural areas because they are often located sparsely throughout the service region, which typically requires these individuals to travel longer periods of time and greater distances across counties spanning larger geospatial areas [4,10].

### 1.2. Transportation and Health

Transportation for access to healthcare-related services is a critical component for maintaining high levels of health and well-being among middle-aged and older adults. Approximately 3.6 million community-dwelling American adults fail to receive healthcare due to a lack of transportation [11]. This is especially true among individuals who are older, female, and those who report multiple chronic conditions and mobility limitations. This is also a barrier for those living in rural communities. While individuals overwhelmingly prefer to transport themselves to and from medical services using their personal automobiles, their ability to do so gradually reduces alongside progressing age-related disease symptomology and other forms of physical and mental impairment [12]. The availability of non-emergency medical transportation (NEMT) may potentially offset negative health ramifications by affording aging adults with transportation to access to non-emergency medical appointments (e.g., medical follow-ups, having a prescription filled), facilitating access to prescription medications, and facilitating participation in therapy-related sessions [12].

Older adults in rural communities have unique transportation barriers to accessing medical care, which include a lack of mass transit options and considerable distances to health-related services. These barriers may prevent many older adults from obtaining needed healthcare (e.g., preventive screenings and routine interactions with healthcare providers) and exacerbate negative health outcomes associated chronic conditions. The length of distance traveled is logically greater among NEMT clients in rural areas but is further extended by the lack of specific specialized medical procedures typically found healthcare settings in urban/suburban areas (e.g., physical rehabilitation, dialysis and mental health services).

**Figure 1:**
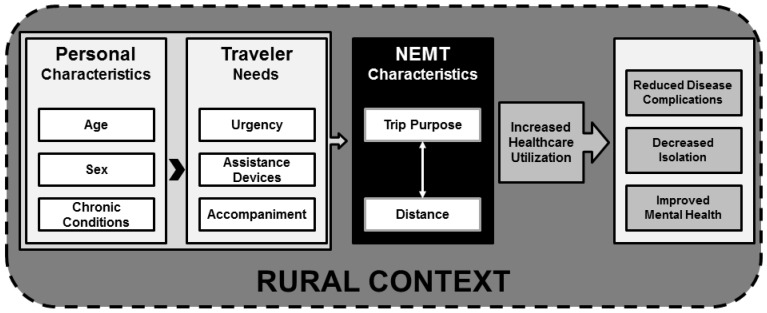
Relationship between personal characteristics, traveler needs, non-emergency medical transportation, and theorized health outcomes in a rural context. NEMT: non-emergency medical transportation.

In a natural experiment of transportation brokerage service implementation in Georgia and Kentucky, healthcare utilization improved and medical expenditures and hospital admissions decreased for diabetic adults [13]. As a result of receiving NEMT services, it is reasonable to believe healthcare utilization will be increased, which may have positive impacts on disease complications/progression, isolation, and mental health status. Figure 1 provides a conceptual framework for understanding the characteristics of NEMT utilization and the contextual factors associated with NEMT use and potential outcomes. This study examines the antecedent factors associated with NEMT use (purpose and distance) across urban and rural settings.

Medicaid provides health insurance to 16% of the rural population [14]. Medicaid also provides subsidized NEMT for access to healthcare for populations with transportation barriers. The purpose of the present paper is to differentiate the characteristics of Medicaid NEMT use, noting the differences between rural and urban trips. To these authors’ knowledge, few (if any) studies have examined these differences among those with equal access to healthcare in terms of both cost and transportation. Findings from this study can help inform healthcare delivery and service improvements for populations who are eligible and who need and utilize this program. Patients who use Medicaid NEMT may be different from those who are eligible but do not use this service. Therefore, the transportation needs of Medicaid rural middle-aged and older adults in Delaware are not completely described in the present paper. This paper focuses on the most vulnerable population accessing ambulatory care.

## 2. Materials and Methods

### 2.1. Study Participants and Procedures

Funded by the U.S. Department of Transportation Federal Transit Administration through cooperative agreements with Easter Seals Project ACTION (ESPA) and the National Center on Senior Transportation (NCST), this study resulted from extensive discussions between (ESPA), the American Medical Association (AMA), and LogistiCare regarding the role of transportation for non-emergency medical care [15]. Monies were awarded to these entities because of their status as training and technical assistance centers that support the: (1) expansion of accessible transportation for individuals of all ages with disabilities; and (2) increase of transportation options for older adults. Activities associated with this study were specifically funded by Easter Seals and utilized information obtained from LogistiCare to develop a greater comprehension about relationships that exist between transportation and healthcare access. LogistiCare is the largest NEMT broker in the USA; active in 39 states and brokering over 26 million NEMT trips per year [16].

For this grant initiative, data were compiled from five states (i.e., Delaware, Nevada, Mississippi, Oklahoma, and Virginia) who met the criteria of having at least five years of complete LogistiCare NEMT utilization data. All users of transportation services brokered by LogistiCare were required to be enrolled in the program to obtain this service. LogistiCare collects comprehensive information for all enrolled riders after eligibility is verified and updates the information as needed. Once potential riders are enrolled for transportation services, LogistiCare collects data for each requested trip including information about the rider, pick-up/drop-off locations, trip purposes, cancellations, rider accompaniment, and cost. Data about the number of calls made by each user to schedule trips were collected. For each ride requested, LogistiCare applied a cost structure based on characteristics of the trip, needs of the rider, whether or not fees were paid to service providers, travel distances, and transport times. Trip-based costs were then linked to individual rider information. Delaware data were selected for preliminary analyses in which construct measures and inter-state comparisons could be drawn. This study exclusively used de-identified data (i.e., no names or addresses) for Medicaid-eligible Delaware members who scheduled a transportation appointment with LogistiCare.

The database was structured as one record per leg for a single rider. Data were summarized at the trip-level (user-trip date). Trips with more than two legs were excluded as there was concern that these trips may differ from one-way or 2-leg round-way trips or that they may represent duplicate scheduled legs. Trips with two legs represented 90.6% of all trips in the dataset (i.e., 5.2% were 1-leg trips, 1.7% were 3-leg trips, 2.3% were 4-leg trips, and 0.2% were trips with 5+ legs). There were 163,277 records for adults aged 45 years and older included in the analysis. Institutional Review Board approval was granted from all involved institutions for this secondary, de-identified data analyses.

### 2.2. Data and Measures

#### 2.2.1. Dependent Variable

The dependent variable for this study was the rurality of participants’ residence. This variable was created using the ZIP Code Tabulation Area (ZCTA) data obtained from the US. Census Summary File 3 2010. Rurality was defined as follows: urban areas included those areas where at least 51% of the ZCTA was defined as a core census block group with a population density of at least 1000 people per square mile and surrounding blocks having an overall density of at least 500 people per square mile (scored as 0) [17]. Rural areas included those areas where at least 51% of the ZCTA was not defined as a core census block group with a population density of at least 1000 people per square mile and surrounding blocks having an overall density of at least 500 people per square mile (scored as 1).

#### 2.2.2. Participant Needs

To measure the participants’ special needs during non-emergency transport, data pertaining to urgency, required assistance devices, and accompaniment were included in analyses. Urgency was identified if at least one leg of the round-trip was scheduled within 24 h of desired pick-up (i.e., scored 0 for no, 1 for yes). The need for assistance devices was determined if the traveler required no assistance (scored 0), a wheel chair (scored 1), or a stretcher (scored 2) during at least one leg of the round-trip transport. Accompaniment by another person during the non-emergency transport was determined if the participant traveled alone (scored 0) or requested to be escorted by an adult (scored 1), child (scored 2), or an appointed personal care assistant (scored 3). 

#### 2.2.3. Trip-Related Characteristics

Logistics of the trip itself, including trip purpose and total trip distance, were included to assess differences between rural and urban trips. Primary purposes in which participants requested non-emergency transportation included dialysis, routine doctor visits, substance abuse-related appointments, mental health-related appointments, medical specialist visits, rehabilitation, and testing/screening-related appointments. This list of purposes represented over 92.6% of all trips utilized. Other trip purposes were omitted from analyses based on frequency distributions and trip purpose. Omitted trip purposes included categories such as adult daycare, wound care, pharmacy, and prosthetic services. Total trip distance was documented for each round-trip, which included the number of miles traveled between pick-up and drop-off. 

#### 2.2.4. Sociodemographics

Personal characteristics of the participants included age groups (i.e., 45 to 54 years, 55 to 64 years, 65 to 74 years, 75+ years) and sex.

### 2.3. Statistical Methods

The data analyzed in this study were inherently correlated since there were multiple trips per patient which occurred over time. To model this form of correlated longitudinal data, we used generalized estimating equations (GEE) with a logit link function. The GEE model was flexible enough to handle correlated data by invoking a user-defined working correlation matrix in the dataset. When the underlying correlation structure between the observations is known, GEE can produce model-based standard errors that are efficient. When the underlying correlation structure is unknown (more likely), GEE can produce empirical estimators which are consistent even when the correlation matrix is incorrectly specified [18,19]. GEE functions as a marginal model by estimating population-averaged effects from selected covariates instead of subject-specific correlation [20]. The latter is often modeled with mixed-effects models. When the need arises to model subject-specific correlation, the mixed-effects model generally employs additional random effect variables which sets it apart from a traditional GEE model which is only concerned with modeling a random intercept. As noted by Zeger and colleagues [20] the GEE approach is better suited to epidemiological research questions where the objective is to interpret coefficients as average effects for the population, not subject-specific changes over time. In the context of this study there was not an explicit focus on how variables effecting the location of a trip pick-up (i.e., rural location) changed over time by patient. The study objective was to recognize the influence of the independent variables on whether a trip originated from a rural location, but in the context of acknowledging a given trip may have been one of many to occur over time for the same patient. This slight but important difference supported our decision to use GEE.

The working correlation matrix used in the GEE model was set to an assumed structure of independence. This was done for two reasons. First, the independence matrix was deemed a good place to begin hypothesizing about the underlying correlation in the data. This structure uses the classic ordinary least squares assumption that observations are independent. Since the correlation structure was unknown, the strategy was to begin with the independence structure assumption and if necessary, progress to additional structure assumptions such as an exchangeable matrix or an unstructured matrix. If the independence structure appeared to be the best fit for the working correlation matrix, then the GEE model would essentially become an independent estimating equation model which Zeger reminds us can be very functional if there is no significant correlation between subjects in the data and if empirical standard errors are used to guard against losses in efficiency [20,21]. GEE model selection was determined by minimizing the quasi-likelihood information criterion (QIC) statistic, a common practice in longitudinal research [22].

## 3. Results

Table 1 displays the sample characteristics based on participant pick-up locations for the first leg of their 2-leg round-trip by rural/urban setting. Of the 163,277 2-leg round-trips, 16.9% originated in areas classified as rural and 57.2% were for female travelers. Approximately 40% of 2-leg round-trips were for travelers aged 45–54 years, 29.1% aged 55–64 years, 16.7% aged 65–74 years, and 14.6% aged 75 years and older. Of the 2-leg round-trips included in this study, the leading four primary trip purposes were for dialysis (50.4%), doctor visits (15.3%), substance use (14.7%), and mental health (8.1%). Over 77% of round-trips were for travelers required no assistance devices on either leg of the transport, 18.4% required a wheelchair, and 3.9% required a stretcher. Further, 5.3% of round-trips included travelers accompanied by others (4.1% accompanied by an adult, 0.4% by a child, and 0.7% by an attendant), and 0.2% of trips were categorized as urgent (i.e., scheduled within 24 h of pick-up). On average, transports were 15.09 (±20.32) miles per round-trip.

When comparing round-trip sample characteristics by rurality of pick-up location, a significantly larger proportion of transports originating from rural locations included travelers who were older (χ^2^ = 1227.87, *p* < 0.001) and female (χ^2^ = 568.53, *p* < 0.001). A significantly smaller proportion of round-trips originating from rural areas transported individuals needing assistance devices (χ^2^ = 350.19, *p* < 0.001); whereas, a significantly larger proportion of round-trips originating from rural areas transported individuals requiring accompaniment by others (χ^2^ = 128.97, *p* < 0.001) and trips for the primary purpose of dialysis (χ^2^ = 3909.14, *p* < 0.001). On average, round-trips originating from rural areas were significantly longer distances (25.41 miles or 40.89 km), relative to trips originating from urban areas (13.0 miles) (*t* = −74.51, *p* < 0.001).

Table 2 displays the results of the logistic regression analysis explaining factors associated with participants being picked-up from a rural location (i.e., being picked up in an urban area served as the referent group or comparator). Relative to those picked-up in urban areas, those picked up in rural areas were transported for round-trips consisting of significantly more miles (OR = 1.03, *p* < 0.001). Compared to those aged 45–54, individuals aged 75 and older were significantly more likely to have been picked up at a rural location (OR = 1.59, *p* < 0.05). Participants who were accompanied by a personal care assistant were significantly more likely to have been transported from a rural location, relative to their counterparts with no accompaniment (OR = 2.57, *p* < 0.001). Conversely, participants needing wheel chair assistance were significantly less likely to have been picked up in a rural location, when compared to those not requiring an assistance device (OR = 0.52, *p* < 0.001). Compared to those whose primary purpose of transportation was dialysis, individuals transported for doctor visits (OR = 0.59, *p* < 0.001), substance abuse (OR = 0.12, *p* < 0.001), medical specialists (OR = 0.58, *p* < 0.001), rehabilitation (OR = 0.59, *p* < 0.05), and testing/screening (OR = 0.68, *p* < 0.05) were significantly less likely to have been picked up at a rural location.

## 4. Discussion

Focusing on the most vulnerable segment of the population (i.e., low income older adults with transportation barriers to healthcare), this is one of the few studies to differentiate the healthcare transportation characteristics by rurality, noting that rural travelers tend to travel longer distances resulting in higher costs. It was expected that transportation and health insurance, as enabling factors, may reduce individual and healthcare characteristics differences previously observed between rural and urban patients. However, the results of this research indicate that differences in patient mix and the type of healthcare utilized exists, suggesting that rural patients may have special needs as they tend to be older and use accompaniment than those needing transportation for shorter urban trips. Understanding the differences in NEMT use by middle and older age by rurality can assist in planning for mobility and healthcare delivery over the life course and by context. For example, reduced support for preventive care in rural areas can end up being costly long-term when accounting for the transportation and treatment of avoidable chronic conditions.

Within this exploratory study, significant differences were observed between NEMT service characteristics for clients in rural versus urban areas expanding upon previous literature documenting rural healthcare differences due to transportation. Consistent with previous studies, results indicate that older age and the need for accompaniment from personal care assistants were more common among participants picked-up in rural areas. Alternatively, wheel chair services and NEMT for purposes other than dialysis were less common among individuals picked-up from rural locations. For example, as previously documented, individuals transported for doctor visits, substance abuse, medical specialists, rehabilitation, and testing/screening were significantly less likely to have been picked up at a rural location. Not surprisingly, longer NEMT trip durations were significantly longer for individuals transported to and from rural destinations, as found in other studies [23,24,25,26].

While this study did not evaluate treatment need, the underutilization of services, or the presence and quality of healthcare facilities, there may be differential delays in routine healthcare and substance use treatment based on perceived and observed barriers in rural areas. For example, NEMT for substance use treatment was less common for the rural patients in this study. Substance use problems are not confined to urban areas; however, additional barriers in accessing treatment may exist. The percent of hospitals that offer substance use treatment services is lower in rural areas (11% vs. 27% in urban areas) and there is less federal funding in rural areas for substance use services. Further, there may be more stigma associated with seeking substance use treatment in rural areas [10]. 

### 4.1. Limitations

We must acknowledge limitations associated with this preliminary study. First, this study may not be generalizable to rural areas in other regions in the U.S. We acknowledge that the definition of rural used in the current study may be viewed as non-urban or semi-rural relative to areas with less dense populations per square mile. We acknowledge this definition did not fully encompass the various levels of rurality, which warrants future investigation using different rural criteria and coding schemes [27]. However, Delaware, a diverse state, represented an interesting opportunity to investigate these issues. This study of Medicaid Delaware middle-age and older adults where patients had equal access to healthcare in terms of cost and transportation, adds to the knowledge that patient and healthcare patterns may differ by place. We acknowledge that Medicaid patients may represent a more homogenous group compared to the larger population. As such, it should be recognized that individuals utilizing subsidized transportation may differ to those who are paying for NEMT services, thus highlighting opportunities for further investigation. It is also important to note that people may select to live in locations based on their health status and may have different opportunities for health based on where they live.

Second, it is important to note that the analyses are somewhat limited as complete health resource utilization and status was not available. Not knowing this information hindered our ability to accurately assess the participants’ true health status, functionality, or the severity/urgency associated with the NEMT trip purpose. Further, differential delays in seeking care based on urban and rural location could not be assessed. It is possible that patients in rural areas experience the longer distance and/or the healthcare options in rural areas as an additional barrier, resulting in less preventive care utilization.

Third, enabling factors about one’s living situation or caregivers were not available. For example, data were not available about the relationship of the person accompanying the NEMT rider, so we could not make inferences about their role during transportation. For example, an adult accompanying the NEMT rider could have been their spouse, friend, co-worker, caregiver, or child. Family and community resources may be important factors for healthcare utilization [28,29].

Fourth, while our dataset was structured so that each record represented one leg for a single rider, it is possible that riders could have been transported in a paratransit vehicle with another rider or multiple riders. This caveat should be acknowledged because having additional riders in the same vehicle may increase the travel time (multiple pick-up and drop-off locations) and decrease the overall miles to healthcare services because a single vehicle with multiple riders may travel the same distance as multiple vehicles transporting a single rider each. These points may be especially salient in rural areas where travel distances between homes and to services are greater.

Finally, the time in which a participant enrolled for LogistiCare services in 2010 may have influenced the frequency or amount in which they utilized NEMT services. For example, if one participant enrolled for services in February and another participant enrolled in October, it is reasonable to assume that the participant enrolling in February had more opportunities to utilize NEMT services. Conversely, if both participants utilized NEMT services 12 times each (i.e., over 11 months and 3 months, respectively), the current study was unable to assess reasons behind the rate of transportation. This is synonymous with the statistical modeling approach taken in this study. We chose to model marginal changes to population averages versus subject-specific change. This is because our research question was based on population health, not on trip-to-trip changes in the independent variables for each patient.

### 4.2. Implications for Research

Given the findings and limitations, there are avenues for future research among patients who have subsidized transportation for healthcare. First, among Medicaid middle-age and older adults, rural trips tended to be longer, and a higher proportion of rural trips were for dialysis. It would be interesting to better understand if perceived or actual travel distance is a barrier for preventive care and whether this contributes to the onset of chronic conditions like end stage renal disease. There is research to demonstrate that travel distance to healthcare is associated with negative health outcomes [30,31]. Other aspects of community health have focused on food deserts. Novel efforts to improve “distance” to healthcare have included telemedicine, mobile clinics, and community design [32].

Second, In the U.S., transportation costs for travel to dialysis facilities has been estimated at more than $3 billion [33]. A study of health expenditure data determined that NEMT for chronic conditions that can lead to more serious conditions (i.e., NEMT for diabetes and hypertension) were determined to be cost saving to highly cost-effective. NEMT for breast cancer screening and colorectal screening were assessed to be moderately cost-effective [34]. However, the U.S. is undergoing political (e.g., healthcare reform), population (e.g., sociodemographic), and physical (e.g., urban sprawl) changes. There is a need to better understand how these changes will impact NEMT utilization and costs. By linking NEMT data to Census, medical, and other data, some complex conditions could be forecasted. 

Third, accompaniment is more common for rural trips in the present study. Additional research including surveys and focus groups can assist in better understanding the role of accompaniment and other ways to reduce barriers to NEMT use.

### 4.3. Implications for Practice

Findings from this study have practical implications for policy makers and individuals in the community striving to overcome rural-based health disparities. While brokered NEMT is available in rural communities, these services may be underutilized by eligible individuals [35], and we observed differences in the type of healthcare utilized by rurality (although we are not completely able to control for the differences in health status between rural versus urban locations) Awareness-raising initiatives should be created in rural communities to educate residents about eligibility requirements and provide assistance to those unfamiliar with how to register for NEMT. However, the availability of NEMT may not be enough to promote health among rural communities. 

Further, the economics of rural healthcare also play a role in identifying available options for linking those who need healthcare services to the best care to meet their diverse needs. Several factors impact the economics of rural health. For example, population density being lower in rural areas may lead to fewer patients for a particular clinic and thereby disproportional healthcare costs (e.g., diseconomies of scale) [36,37]. In addition, rural areas also face disproportional poor health [10,38,39], are commonly underserved (e.g., Health Professional Shortage Areas) [40], and face gaps in access to health-related services (e.g., longer distances, poorer quality) [41,42] relative to urban areas. In addition, the relative population density and larger service areas for emergency medical services (EMS) in rural areas may contribute to higher cost-per-transport that that seen in urban areas [36,43]. This is further complicated by the already existing racial and ethnic healthcare disparities facing rural America [44]. Given broad factors associated with higher cost for some services, greater distance to certain care services, and the diversity in rural areas (e.g., demographic, economic sectors) [45] it is difficult to determine broad decisions on whether to provide a greater diversity of services within rural areas or whether to transport individuals from rural areas to certain healthcare services they need.

To complement existing NEMT, initiatives to develop community capacity should be created to offer medical-related screening and treatment services closer to areas in which older Americans with chronic diseases reside. Such initiatives would rely on expanding the service infrastructure’s capacity to provide services in Health Professional Shortage Areas (HPSA) where residents may experience greater gaps in access to healthcare and poorer health than those in non-HPSA [46]. Bringing services closer to the community may reduce barriers associated with farther travel distances. To increase success when initiating such community capacity efforts, mobile service coordinators should solicit input and feedback from key community leaders and demographics to ensure proposed strategies reflect the true needs of the community and are framed within an appropriate context [47]. 

As another strategy to improve health status and reduce the need for healthcare utilization, communities are encouraged to embed evidence-based programs into existing organizations in rural communities (e.g., faith-based organizations, senior centers, residential facilities) through the aging services network and public health system [24]. Offering prevention programs such as those focusing on chronic disease self-management, fall prevention, and physical activity within this service infrastructure brings these services closer to participants’ homes and has potential to reduce travel-related attrition due to distance [25]. Further, these programs have shown to reach diverse populations [23,26], improve health status indicators [48,49], and increase physical functioning and mobility [50]. 

## 5. Conclusions

Transportation to NEMT serves a critical role for millions of Americans, especially those in rural areas where distance to providers may serve as an additional barrier to healthcare access. Understanding the contextual factors associated with utilization of NEMT allows for a more complete picture in understanding utilization of NEMT for specific groups (e.g., rural residents). This study serves a critical role in closing the gap in what is known about differences for rural and urban Medicaid beneficiaries utilizing NEMT. More research is needed to improve our understanding of the potential benefits of NEMT and community capacity in rural areas across more vulnerable populations (e.g., minority, rural remote residents).

## Figures and Tables

**Table 1:** Sample characteristic by rurality of pick-up location.

	Total (*n* = 163,277)	Urban (*n* = 135,759)	Rural (*n* = 27,518)	χ^2^ or *t*	*p*
Age: 45–54	39.6%	41.1%	32.2%	1227.87	<0.001
Age: 55–64	29.1%	29.1%	28.8%		
Age: 65–74	16.7%	16.2%	19.1%		
Age: 75+	14.6%	13.5%	20.0%		
Male	42.8%	44.1%	36.3%	568.53	<0.001
Female	57.2%	55.9%	63.7%		
Non-Urgent Request	99.8%	99.8%	99.7%	2.52	0.112
Urgent Request	0.2%	0.2%	0.3%		
Assistance Device: None	77.6%	76.8%	81.9%	350.19	<0.001
Assistance Device: Wheel Chair	18.4%	19.2%	14.7%		
Assistance Device: Stretcher	3.9%	4.0%	3.4%		
Accompaniment: None	94.7%	95.0%	93.5%	128.97	<0.001
Accompaniment: Adult	4.1%	3.9%	5.2%		
Accompaniment: Child	0.4%	0.4%	0.3%		
Accompaniment: Personal Care Assistant	0.7%	0.7%	1.0%		
Trip Reason: Dialysis	50.4%	48.5%	60.0%	3909.14	<0.001
Trip Reason: Doctor Visit	15.3%	15.0%	16.5%		
Trip Reason: Substance Abuse	14.7%	17.1%	2.9%		
Trip Reason: Mental Health	8.1%	8.1%	8.3%		
Trip Reason: Medical Specialist	6.3%	6.1%	7.5%		
Trip Reason: Rehabilitation	3.5%	3.6%	3.0%		
Trip Reason: Testing/Screening	1.6%	1.6%	1.8%		
Total Miles *	15.09 (±20.32)	13.00 (±18.14)	25.41 (±26.42)	−74.51	<0.001

* means and standard deviations are presented for Total Miles.

**Table 2:** Factors associated with rural pick-up for non-emergency medical transportation.

					95% CI of β		95% CI of OR	
	β	OR	S.E.	*p*	Lower	Upper	Lower	Upper
Age: 45–54								
Age: 55–64	0.14	1.15	0.18	0.444	−0.22	0.50	0.80	1.65
Age: 65–74	0.23	1.26	0.23	0.311	−0.21	0.67	0.81	1.95
Age: 75+	0.47	1.59	0.22	0.038	0.03	0.91	1.03	2.47
Male								
Female	0.29	1.33	0.16	0.074	−0.03	0.61	0.97	1.83
Urgent Request	−0.04	0.96	0.43	0.922	−0.88	0.79	0.42	2.21
Assistance Device: None								
Assistance Device: Wheel Chair	−0.65	0.52	0.18	<0.001	−1.01	−0.29	0.36	0.75
Assistance Device: Stretcher	−0.58	0.56	0.32	0.068	−1.21	0.04	0.30	1.04
Accompaniment: None								
Accompaniment: Adult	0.27	1.31	0.15	0.068	−0.02	0.56	0.98	1.75
Accompaniment: Child	−0.25	0.78	0.34	0.476	−0.92	0.43	0.40	1.54
Accompaniment: Personal Care Assistant	0.94	2.57	0.23	<0.001	0.50	1.39	1.64	4.01
Trip Reason: Dialysis								
Trip Reason: Doctor Visit	−0.53	0.59	0.14	<0.001	−0.79	−0.26	0.45	0.77
Trip Reason: Substance Abuse	−2.14	0.12	0.53	<0.001	−3.18	−1.11	0.04	0.33
Trip Reason: Mental Health	−0.40	0.67	0.28	0.152	−0.94	0.15	0.39	1.16
Trip Reason: Medical Specialist	−0.55	0.58	0.15	<0.001	−0.85	−0.25	0.43	0.78
Trip Reason: Rehabilitation	−0.54	0.59	0.24	0.027	−1.01	−0.06	0.36	0.94
Trip Reason: Testing/Screening	−0.39	0.68	0.16	0.014	−0.70	−0.08	0.50	0.92
Total Miles	0.03	1.03	0.00	<0.001	0.02	0.03	1.02	1.03
